# New Jersey State Homœopathic Medical Society

**Published:** 1888-06

**Authors:** 


### The New Jersey State Homœopathic Medical Society.

This pamphlet of one hundred and thirty-two pages contains a selection of papers read at various times by members of this Society. It would, perhaps, be an improvement if all Committees on Publication exercised a wise discretion as to the matter published; many useless papers are published under the usual system of printing any and every paper offered.

